# Lymph-Derived Neutrophils Primarily Locate to the Subcapsular and Medullary Sinuses in Resting and Inflamed Lymph Nodes

**DOI:** 10.3390/cells10061486

**Published:** 2021-06-12

**Authors:** Jenny de Castro Pinho, Reinhold Förster

**Affiliations:** 1 Institute of Immunology, Hannover Medical School, 30625 Hannover, Germany; jennypoetzsch@gmail.com; 2 Cluster of Excellence RESIST (EXC 2155), Hannover Medical School, 30625 Hannover, Germany

**Keywords:** neutrophils, lymphatics, lymph node, two-photon imaging

## Abstract

Neutrophils are the first immune cells to be recruited from the blood to the tissue site of an infection or inflammation. It has been suggested that neutrophils are capable of migrating from the infected tissue via lymphatic vessels to the draining lymph nodes. However, it remains elusive as to which areas within the lymph nodes can be reached by such reversely migrating cells. To address this question, we applied a model for adoptive neutrophil transfer into the afferent lymphatic vessel that drains towards the popliteal lymph node in mice. We showed that resting and in vitro-activated neutrophils did not enter the lymph node parenchyma but localized primarily in the subcapsular and medullary sinuses. Within the medulla, neutrophils show random migration and are able to sense laser-induced sterile tissue injury by massively swarming to the damaged tissue site. Co-injected dendritic cells supported the entry of resting neutrophils into the lymph node parenchyma via the subcapsular sinus. In contrast, in vivo-activated adoptively transferred neutrophils were capable of migrating into the interfollicular areas of the lymph node. Collectively, the data presented here give further insights into the functional behavior of neutrophils within the lymph nodes.

## 1. Introduction

For the defense of invaded pathogens, neutrophils are rapidly recruited from the blood to infected tissue sites. Granulocytic neutrophils are the first immune cells arriving at the site of infection via postcapillary venules [1]. The migration of neutrophils towards the infected or inflamed site within the tissue can be divided into different phases [2,3]. Initially, neutrophils roll along the vessel wall, followed by their attachment to the endothelial cells, subsequent neutrophil arrest and crawling, before they transmigrate through the vessel wall into the tissue [1].

For a long time, it had been accepted that neutrophils undergo apoptosis at the site of infection and get cleared by macrophages. However, evidence is accumulating that a considerable proportion of the neutrophils that entered inflamed tissue sites are capable to migrate back to the blood circulation [2]. This reverse migration is usually mediated by chemo-attractants [2]. There is evidence that Leukotriene B4 (LTB_4_) is not only involved in the migration towards the tissue but also in the reverse migration out of the tissue [2]. Likewise, the atypical receptor ACKR1 was shown to be involved in this process [4].

In addition to migrating back to blood circulation, it was also suggested that neutrophils can exit from inflamed sites via afferent lymphatic vessels [2]. The presence of neutrophils inside lymphatic vessels was shown earlier by microscopy, indicating the migration of neutrophils via lymphatic vessels towards draining lymph nodes [5,6,7,8]. Additionally, it was shown that neutrophil migration was restricted to the ipsilateral lymph nodes draining the site of infection but not to the contralateral site. These observations favor the hypothesis that neutrophils enter lymph nodes via lymphatics, rather than blood vessels [5,7,9]. In contrast, recent studies excluded neutrophil lymph node entry via lymphatics, as neutrophils were only found within blood but not lymphatic vessels [10].

In general, neutrophil reverse migration via lymphatics has not been unambiguously characterized. There is evidence that neutrophil migration from the inflamed tissue to the draining lymph node is dependent on the chemokine receptor CCR7 [11]. In that model CCR7 on neutrophils recognizes high levels of the chemokines CCL19 and CCL21, which are produced by fibroblastic reticular cells inside the T cell zone of the lymph node [3,11]. In contrast, Hampton and colleagues showed lately in an *S. aureus* infection model that neutrophils are capable of migrating to the lymph node independent of CCR7 [6,12]. A recent study could confirm CCR7-independent neutrophil migration to draining lymph nodes during *S. aureus* infection [10]. The model of a CCR7-independent migration of neutrophils from the infection site via afferent lymphatics to the draining lymph node seems rather plausible since this chemokine receptor is hardly expressed on any neutrophil subset [13]. Furthermore, CD11b (Integrin α-M) and the chemokine receptor CXCR4 were recently suggested to contribute to neutrophil lymph node homing via lymphatics [6,12]. Blocking of CD11b or CXCR4 [13] resulted in lower neutrophil numbers in the draining lymph nodes upon *S. aureus* infection [6].

To circumvent the requirements that control neutrophil lymphatic entry at peripheral tissue sites we adoptively transferred neutrophils by intra lymphatic injection into the afferent lymphatic vessel that drains towards the popliteal lymph node in mice. We show that both resting as well as activated neutrophils largely locate to the medullary sinuses and are largely excluded from the deep T cell zone. However, when delivered together with dendritic cells neutrophils get access into the interfollicular area.

## 2. Materials and Methods

### 2.1. Mice

C57BL/6 N Crl (C57BL/6; Charles River, (Sulzfeld, Germany) were kept in the central animal facility at Hannover Medical School under specific pathogen-free conditions and were used at the ages of between 7 and 14 weeks. All animal experiments were approved by the Niedersächsisches Landesamt für Verbraucherschutz und Lebensmittelsicherheit (LAVES; 33.12-42502-04-17/2660; 24 November 2017).

### 2.2. Immunization

In immunization and infection models, mice were subcutaneously (s.c.) injected with different pathogens or pathogen-derived components, to induce an immune response in the popliteal lymph node. Mice were anesthetized with a single intraperitoneal injection of 50 mg/kg body weight Ketamin and 10 mg/kg body weight Xylazin, and pathogens were s.c. injected in the footpad of the hind leg using a volume of 30 µL PBS four hours to 1 day before intra-lymphatic (i.l.) cell delivery. Per s.c. footpad injection 10^7^ CFU of heat-inactivated Pseudomonas (*P.*) *aeruginosa*, or 1 × 10^6^ IU MVA (Modified Vaccinia virus Ankara–Virus) were applied. Lipopolysaccharide (LPS) was injected s.c. at a concentration of 0.3 mg/kg body weight in 30 µL PBS to activate lymph nodes. In other experiments, 5 mg/kg body weight of the tripeptide fMLP (N-Formylmethionyl-leucyl-phenylalanine) were used for the s.c. injection. One day before cell isolation, donor mice were intranasally infected with 1 × 10^8^ IU MVA for the generation of in vivo-activated neutrophils.

### 2.3. Intra-Lymphatic Injection

For the i.l. cell transfer, mice were anesthetized by intraperitoneal injection of 100 mg/kg body weight Ketamin and 10 mg/kg body weight Xylazin. Subsequently, hind legs were shaved and a short longitudinal skin incision above the vena saphena allowed the visualization of both lymphatic vessels left and right to the vena saphena. Borosilicate glass capillaries (outer diameter: 1.5 mm; inner diameter: 1.17 mm; Harvard Apparatus, Holliston, MA, USA) were pulled either manually or with the P-1000 Flaming/Brown micropipette puller (Sutter Instrument, Novato, CA, USA), and ground using the EG-44/EG-45 micropipette grinder (Narishige, London, UK). Cell suspensions with a volume of 5 to 10 µL (50,000–75,000 cells) were injected at a maximum pressure of 35 kPa in pulses of 90 to 120 s. The injection of neutrophils was performed using kininogen-coated glass capillaries (10 µg/mL) [14]. For the injection procedure, a PLI-100/PLI-100A microinjector (Harvard Apparatus), as well as the micromanipulator (MN-151; Narishige) to stabilize the glass capillaries, were used. During the entire surgical procedure, skin incisions were kept moist by application of PBS. Mice were monitored and kept warm to preserve a normal body temperature.

### 2.4. Isolation of Neutrophils

Neutrophils were isolated from the bone marrow of untreated mice or from the blood and lungs of the MVA-infected mice. Femur and tibia of the murine hind legs were removed, the ends were cut open and bone marrow cells were harvested by a short centrifugation step (30 s at 5500 rpm). Erythrocytes were lysed for 3 min on ice in Erylysis-buffer (168 mM ammonium chloride, 10 mM potassium bicarbonate and 1.095 mM Disodium ethylenediaminetetraacetate dehydrate in water; pH 7.3). Cells were then separated accordingly using the untouched Neutrophil Isolation Kit. In vivo-activated neutrophils were isolated from the lungs and blood of mice that were intranasally immunized with MVA. Blood was collected in sodium citrate, erythrocytes were lysed and neutrophils isolated by MACS as described above. Lungs were flushed with PBS and digested for 1 h at 37 °C (100 rpm) in red DMEM containing 100 µg/mL Liberase and 0.3 mg/mL DNase. After the lysis of erythrocytes, lung cells were blocked for 15 min on ice with 5% (*v*/*v*) rat serum and 10% (*v*/*v*) of an antibody against CD16 (FcγRIII)/CD32 (FcγRII) (clone: 2.4G2). Next, cells were stained with FITC-conjugated anti-CD11b (clone: M1/70.15; Invitrogen, Waltham, MA, USA) as well as PE-conjugated anti-Ly6G (clone: 1A8; Biolegend, San Diego, CA, USA) for 30–40 min on ice. Subsequently, FITC+ PE+ cells (neutrophils) were sorted using a FACS Aria Fusion at Hannover Medical School Central Cell Sorting Core Facility. Depending on availability, neutrophils were isolated wild-type mice and labeled with fluorescent dyes or from transgenic mice expressing a cyan fluorescent protein (CFP).

### 2.5. Generation of Dendritic Cells

Dendritic cells were generated in vitro from isolated bone marrow cells of the femur and tibia of murine hind legs. Bone marrow cells were isolated as described above. A total of 2.5–3 × 10^6^ bone marrow cells were cultured for 8 days in RPMI with 10% (*v*/*v*) heat-inactivated FCS, 100 Units/mL Penicillin, 100 µg/mL Streptomycin, 2 mM L-Glutamine, 0.00035% (*v*/*v*) β-mercaptoethanol and 5% (*v*/*v*) GM-CSF (from cell culture supernatant) in a 10 cm petri dish. The medium was renewed on days 3 and 6. On day 8, 5 × 10^6^ immature DCs were activated overnight by removing GM-CSF-containing medium and adding fresh medium (without GM-CSF) supplied with 1 µg/mL LPS in a 10 cm cell culture dish for adherent cells.

### 2.6. Neutrophil Activation

MACS purified neutrophils were activated in vitro by incubation with heat-inactivated *P. aeruginosa* in a ratio of 1:3 (neutrophil:bacteria) for 60–120 min at 37 °C to trigger phagocytosis-induced activation. Activated neutrophils were washed to remove free bacteria and re-suspended in PBS/3% (*v*/*v*) FCS. Activated neutrophils were immediately delivered via i.l. injection.

### 2.7. Flow Cytometry

For flow cytometry, antibody staining of isolated cells from organs or cells from cell culture was performed in a volume of 100 µL with a maximum of 750,000 cells per staining reaction. Fc receptors were blocked for 15 min on ice by using 10% (*v*/*v*) of cell culture supernatant, containing an antibody against CD16 (FcγRIII)/CD32 (FcγRII) (clone: 2.4G2) diluted in PBS/3% (*v*/*v*) FCS and 5% (*v*/*v*) rat serum. Then, the antibody mixtures were added and incubated on ice for 20–30 min. Cells were washed once with PBS/3% (*v*/*v*) FCS. Cells were re-suspended in 50–80 µL PBS/3% (*v*/*v*) FCS and analyzed using an LSR II (BD Biosciences). All flow cytometer data files were analyzed with FlowJo7.5 software. The following antibodies were used: anti-CD11b dye (clone M1/70.15; PE, FITC from Invitrogen; clone M1/70; PE-Cy7 from Biolegend, eFl450 from eBioScience, San Diego, CA, USA); anti-Ly6G (clone 1A8; PE from Biolegend); anti-CD62L (clone MEL14; APC from Biolegend); AF488- and Pacific Blue-labelling were homemade.

### 2.8. Labelling of Cells with Fluorescent Cell Dyes

For adoptive transfer, cells were stained with the fluorescent dyes eFluor™ 450 or eFluor™ 670 (Invitrogen). Cells were adjusted to 2 × 10^7^ cells/mL PBS and dye was added at a final dilution of 1.25 µM for 10 min at 37 °C. The labeling reaction was stopped by adding 4–5× volume of ice-cold PBS/10% (*v*/*v*) FCS. In some experiments, cells were labeled with CMFDA (5-chloromethylfluorescein diacetate; Life Technologies, Carlsbad, CA, USA). Approximately 1 × 10^7^ cells/mL medium (e.g., RPMI) was pre-warmed in a water bath at 37 °C, and stained with 0.25 µM CMFDA for 15 min at 37 °C. Cells were washed and re-suspended at defined concentrations in PBS/3% (*v*/*v*) FCS for further use.

### 2.9. MACS

After lysis of erythrocytes, cells were separated according to the manufacturer’s instructions by using magnetic-activated cell sorting (MACS, Miltenyi Biotec, Bergisch Gladbach, Germany) and the “untouched” neutrophil Isolation kit for mice. With this kit, neutrophils were isolated applying the manufacturer’s antibody cocktail that binds to all cells except neutrophils. Next, antibodies bound to cells were linked to magnetic beads which were then separated via special separation columns. During this step, cells bound to magnetic antibodies were retained in the column while untouched neutrophils were able to flow through for collection and further use.

### 2.10. Immunohistology

Lymph nodes were removed from sacrificed mice and fixed in PBS, 2% PFA, 30% Sucrose at 4 °C overnight. Lymph nodes were washed in PBS for 3–10 min, embedded in Tissue Tek O.C.T. (Sakura Finetek, Umkirch, Germany) and sectioned in 8 µm thick slices using a cryotome (Leica CM 3050 S). Cryosectioned slides were stored at −20 °C, or after a short drying time directly used for histology staining. For this staining, slides were rehydrated for 5 min with 1× TBS-T (1 M Tris (Base, Mechelen, Belgium), 1.55 M NaCl; pH 7.5; 0.05% Tween20) and blocked for 15 min with 10% (*v*/*v*) rat serum as well as 10% (*v*/*v*) Fc-Block (cell culture supernatant containing the antibody clone 2.4G2; anti-CD16 (FcγRIII)/CD32 (FcγRII)) in TBS-T. After blocking, slides were stained with one or more of the following antibodies: anti-LYVE-1 (purified polyclonal from Acris followed by anti-rabbit-FITC or Cy5-labelled from Jackson or clone ALY7; eFl660 from eBioScience), anti-IgD (clone: HB250, Cy3-, Cy5- home labelled), anti-CD11b (clone M1/70; eFl450 from eBioScience) or anti-Ly6 G (clone 1A8; PE from Biolegend) for 45 min to 1 h. Cell nuclei were stained using either DAPI (4′,6-diamidino-2-phenylindole, 1 µg/mL, Sigma, St. Louis, MO, USA) or PI (propidium iodide, 10 µg/mL, Fluka, Munich, Germany) for 2 min. Immuno-stained slides were dried overnight in darkness at room temperature.

### 2.11. Fluorescence Microscopy and Analysis of Images

Composite images of lymph node cryosections were taken after immunofluorescence staining with the AxioCam MRm camera (Carl Zeiss, Jena, Germany) connected to an Axiovert fluorescence microscope (Carl Zeiss), using PlanApochromat objectives 10×/0.45 and 20×/0.75 (magnification/numerical aperture). The images were processed with AxioVision 4.8.2 software. Cell counts were performed on 3–4 sections per lymph node using Imaris ×64 8.3.1 (Bitplane, Zurich, Switzerland). In order to obtain the distribution of adoptively transferred neutrophils within the lymph node, neutrophils were allocated to three lymph node compartments (medulla, parenchyma and subcapsular sinus). The definition of subcapsular as well as medullary sinuses in popliteal lymph nodes was performed based on the immunofluorescent staining of LYVE-1. For the neutrophil migration distance measurement, the lymph node borders were outlined manually and neutrophils were semi-automatically tracked using the Imaris spot detection function. Migration distance measurements were performed using the shortest calculated distance of the cell from the subcapsular sinus by using a macro (by Tim Worbs, Institute of Immunology, (Hannover Medical School, Hannover, Germany) for ImageJ. For this, the plugins “analyze particles” and “line graph” from ImageJ were used to examine the coordinates of the manually outlined subcapsular sinus and tracked neutrophils. The macro “Visual Basic for Applications” in Microsoft Excel was used for listing the final distance results of the neutrophils. Migration distances were exclusively obtained from neutrophils that migrated into the lymph node parenchyma.

Two-photon microscopy movies of i.l. injected neutrophils in lymph nodes were acquired using a TriM Scope setup (La Vision Biotec, Bielefeld, Germany) as described [15,16]. After the transfer of neutrophils, mice were killed at different time points before an eFl660-conjugated LYVE-1 antibody (33.34 µg/mL) was injected to distinguish the medullary from the sinus region in the popliteal lymph node. Afterward, excised popliteal lymph nodes were glued into an imaging chamber, and an oxygen-supplied medium circulation with a temperature of 37 °C was connected to mimic natural conditions. In order to excite eGFP or eCFP (enhanced cyan fluorescent protein) neutrophils, Ti:Sa laser was tuned to 920 nm for a good visualization. For the excitation of eFl660, the optical parametric oscillator (OPO) was tuned to 1100 nm. Movies were taken with a scan field range between 300 × 300 µm and 500 × 500 µm for up to 60 min. Data acquired from 2-photon microscopy were analyzed using Imaris ×64 8.3.1 or Imaris ×64 7.7.2 software. All movies were median-filtered.

### 2.12. Statistical Analysis

A statistical significance test was performed for two groups of independent samples. GraphPad Prism4 (GraphPad Software, Inc., San Diego, CA, USA) was used to perform the two-tailed non-parametric Mann–Whitney test assuming no Gaussian distribution. Results with a *p*-value ≤0.05 were considered significant. The following symbols are used * *p* < 0.05; ** *p* < 0.01; *** *p* < 0.001. Data regarding the distribution of cells within the lymph node are represented as mean with SD. Migration distances are shown as the median.

## 3. Results

### 3.1. Purification and Intra Lymphatic Injection of Resting Neutrophils to Non-Inflamed Lymph Nodes

**Figure 1:**
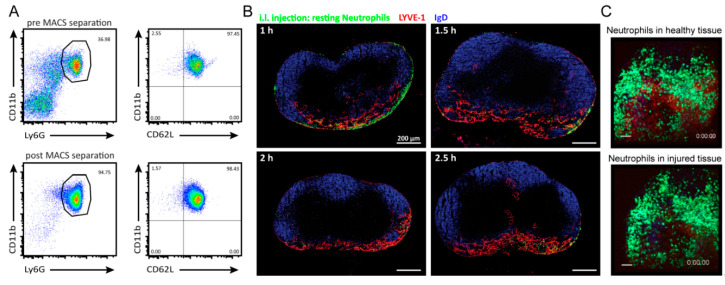
Activation status, positioning and motility of resting bone marrow neutrophils in non-inflamed lymph nodes. (**A**) FACS plots with frequency of CD11b^+^ Ly6G^+^ cells (neutrophils; left plots) as well as the activation status (CD11b^+^ CD62L^+^; right plots) of bone marrow neutrophils before (upper row) and after MACS separation (lower row). Representative plots from more than 10 experiments are shown. (**B**) Fixed histological sections of popliteal lymph nodes 1 to 2.5 h post i.l. transfer of resting bone marrow neutrophils. Sections are stained with anti-LYVE-1 (red) and anti-IgD (blue) antibodies. Injected neutrophils are shown in green. Scale bar 200 µm. For all time points investigated a total of 16 lymph nodes was analyzed. (**C**). (**Upper** part, Supplementary Video S1) This 2-photon video shows the rapid and random migration of neutrophils (green) within the medulla of the popliteal lymph node, possessing an intense anti-LYVE-1 staining (red) as well as the second harmonics signal of collagen (blue). Neutrophils were i.l. injected 4.5 h before imaging. (**Lower** part, Supplementary Video S2) shows rapid and directional migration of neutrophils (green) towards the site of sterile tissue injury induced by laser radiation to a defined tissue area. The lymph node area is identical to the imaged area of Supplementary Video S1. This video shows the medulla using anti-LYVE-1 (red) and dense collagen structures (SHG, blue). Video show 20-sec time intervals with a playback time of 5 fps. Scale bar 50 µm. The movie is a representative 2-photon imaging of the medullary area from a non-inflamed popliteal lymph node 3 h after i.l. injection of resting neutrophils. Neutrophils are depicted in green and the second harmonics signal (SHG) in blue, illustrating the collagen structure of the lymph node. After neutrophil transfer, mice were killed at the respective time point and then an antibody detecting LYVE-1 was injected to visualize lymphatic vessels. The video shows 10-sec time intervals with a playback time of 10 fps (frames per second). Scale bar; 20 µm.

We characterized neutrophils from bone marrow by flow cytometry. Neutrophils are Ly6G^+^ CD11b^+^ and account for approximately 30% to 40% of all nucleated cells in bone marrow (Figure 1A). Applying the “untouched neutrophil isolation kit”, neutrophils were isolated by MACS separation, yielding purities of 90 to 95% (Figure 1A). To test for a potential activation of neutrophils during the separation process, the activation profile of these cells was analyzed by staining with anti-CD11b and anti-CD62L monoclonal antibodies. Activated neutrophils shed CD62L (L-selectin), while expression of CD11b increases. When comparing the expression profile of CD11b and CD62L before and after MACS separation (Figure 1A), no differences could be observed, indicating that the process of separation did not activate neutrophils.

Next, resting neutrophils were used for intra-lymphatic (i.l.) injection to analyze their migration properties to and within lymph nodes. Intra-lymphatically transferred resting bone marrow neutrophils positioned primarily in the medullary region of the lymph node, where Lymphatic Vessel Endothelial Hyaluronan Receptor 1 (LYVE-1^+^) lymphatic endothelial cells are present at high numbers (shown in red in Figure 1B). Early after the transfer, some neutrophils were additionally found in the subcapsular sinus of the popliteal lymph node. The B cell follicles (blue areas in Figure 1B), as well as the T cell zone (not stained on these sections), were not infiltrated by adoptively transferred neutrophils. Interestingly, even 2.5 h after transfer, neutrophils showed a similar positioning within the lymph nodes. In contrast, at that time of transfer, dendritic cells already reached the T cell zone, as reported earlier [17]. The viability of neutrophils after i.l. transfer was revealed by 2-photon microscopy (Figure 1C). As shown in Supplementary Video S1, neutrophils revealed high motility within the medullary area of the lymph node. These results imply that resting neutrophils keep their motility but are not capable to migrate into the parenchyma of non-inflamed lymph nodes.

### 3.2. Positioning and Migration of Resting Neutrophils in Inflamed Lymph Nodes

We tested whether neutrophils might enter the lymph node parenchyma of popliteal lymph nodes that had been activated before with different bacterial components or pathogens.

**Figure 2:**
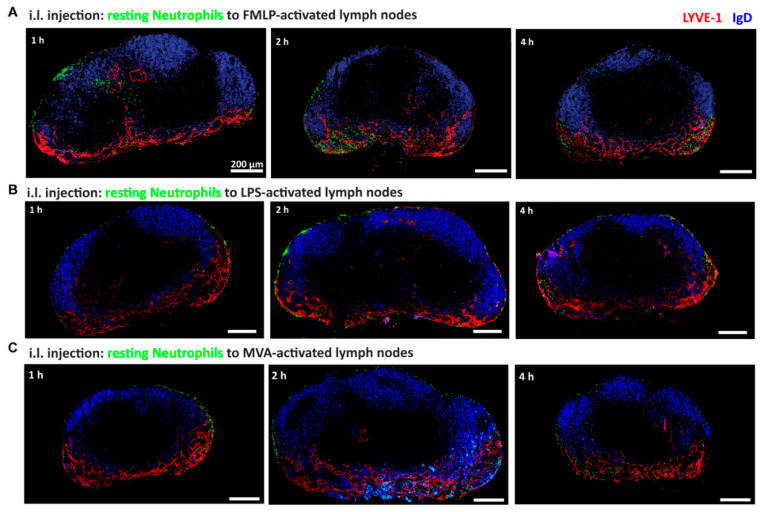
Positioning of resting neutrophils in activated lymph nodes. (**A**) Cryosectioned, fMLP–activated popliteal lymph nodes 1 to 4 h after intra-lymphatic transfer of resting neutrophils (green) are shown. Histological staining with anti-LYVE-1 (red) and anti-IgD (blue). Scale bar 200 µm. For all time points investigated a total of 12 lymph nodes was analyzed. (**B**) Fixed cryosections of LPS–activated (1 day) popliteal lymph nodes 1 to 4 h after i.l. transfer of neutrophils (green), stained with antibodies against LYVE-1 (red) and IgD (blue) are shown. Scale bar 200 µm. For all time points a total of 6 lymph nodes was analyzed. (**C**) Lymph node cryosections show MVA-infected (4 h p.i.) lymph nodes 1 to 4 h post i.l. transfer of neutrophils (green). All sections were stained using anti-LYVE-1 (red) and anti-IgD (blue) antibodies. Scale bar 200 µm. Representative data from 5 analyzed lymph nodes are shown.

Bacterial components, such as peptides, compounds of the bacterial cell wall, flagellin or DNA can be recognized by pattern recognition receptors (PRRs) expressed by neutrophils and are therefore suited to activate these cells. We first transferred neutrophils i.l. and 10 min later applied the bacterial tripeptide fMLP that is known to attract neutrophils s.c. into the footpad of mice. Neutrophils were found to position to the subcapsular sinus as well as the medullary region of the popliteal lymph node (Figure 2A). Occasionally, neutrophils formed cell clusters within the sinus or were able to migrate towards the lymph node parenchyma via an intermediate sinus (Figure 2A, left). Since fMLP is known to rapidly activate neutrophils, we cannot exclude that not only the lymph node but also the neutrophils were activated in this experimental set-up.

LPS is a cell wall compound of Gram-negative bacteria and is recognized by Toll-like receptor 4 (TLR4) on neutrophils [18]. We thus s.c. injected LPS to activate the popliteal lymph node and one day later i.l. transferred neutrophils to study their migration and positioning behavior under these conditions. Adoptively transferred, resting neutrophils localized mainly inside the subcapsular sinus within the first 4 h after transfer (Figure 2B). They were also found in the medullary region of the lymph node, especially 8 h after transfer (data not shown). However, neutrophils were not found in B cell follicles or inside the T cell zone of the lymph node (Figure 2B).

It had been reported that neutrophils migrate to draining lymph nodes upon infection with the poxvirus MVA [19,20]. We therefore infected mice s.c. in the footpad with MVA that leads to activation of the popliteal lymph nodes. Then, 4 h after infection, resting neutrophil were i.l. delivered and their homing was studied. As observed before 1 to 4 h of transfer, neutrophils localized within the subcapsular sinus and the medullary region (Figure 2C).

### 3.3. Positioning and Migration of Activated Neutrophils in Inflamed Lymph Nodes

**Figure 3:**
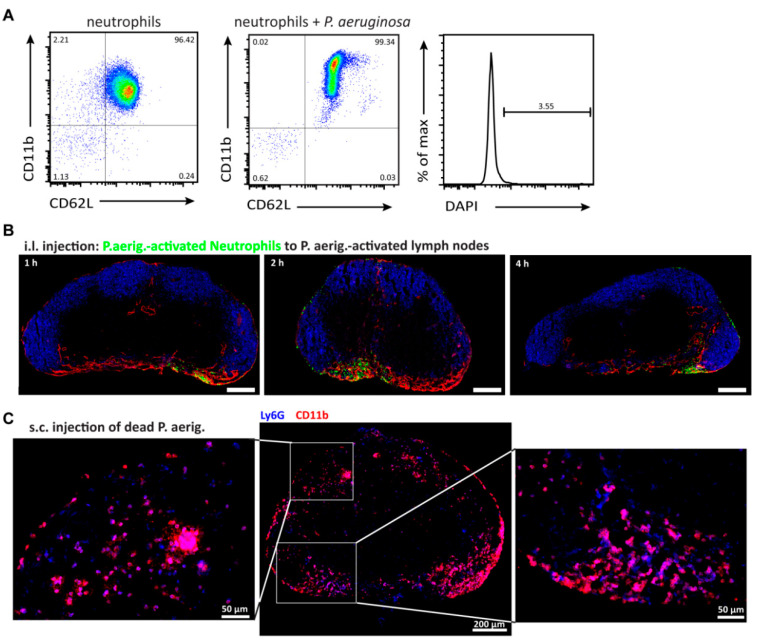
Neutrophil positioning in *P. aeruginosa*–activated popliteal lymph nodes. (**A**) FACS plots showing the activation status of resting neutrophils (**left**) or neutrophils incubated with heat-inactivated *P. aeruginosa* for 60 min (**middle**). The **right** histogram depicts DAPI–positive cells after activation in vitro. Representative plots from 5 independent experiments are shown. (**B**) Popliteal lymph node 1-day post-immunization with heat-inactivated *P. aeruginosa* and 1, 2 or 4 h after i.l. transfer of *P. aeruginosa*-activated neutrophils (green). Lymph node cryosections were stained with the following antibodies anti-LYVE-1 (red) and anti-IgD (blue). Scale bar; 200 µm; a total of 12 lymph nodes was analyzed for all time points investigated. (**C**) An overview of a cryosection from a *P. aeruginosa*–activated popliteal lymph node 1-day post-immunization stained with anti-Ly6G (blue) and anti-CD11b (red) is shown. Twenty× zoom-in pictures are shown for the subcapsular sinus region (left picture) as well as the medullary region (right picture) of the lymph node. Double positive cells (Ly6G^+^ CD11b^+^ in pink) depict infiltrated endogenous neutrophils. Scale bar overview 200 µm; zoom in, 50 µm. Representative data of 3 lymph nodes are shown.

We next addressed the positioning of i.l. delivered activated neutrophils to inflamed lymph nodes. To that end, neutrophils were activated in vitro, by incubation with heat-inactivated *P. aeruginosa* for up to one hour. Compared to incubation with PBS exposure, *P. aeruginosa* led to the upregulation of CD11b, but not to the shedding of CD62L on neutrophils and did not substantially affect their viability (Figure 3A). Activated neutrophils were then i.l. transferred in mice that received an s.c. injection of heat-inactivated *P. aeruginosa* 8 or 24 h earlier. After one hour of i.l. transfer of neutrophils into mice that been s.c. treated with bacterial lysate for eight hours, adoptively transferred cells were found primarily in the subcapsular sinus of the popliteal lymph node (data not shown). In mice where the popliteal lymph node had been pre-activated 24 h, i.l. delivered neutrophils located primarily to the medullary region while some were still present in the subcapsular sinus (Figure 3B). These results indicate that also after in vitro priming neutrophils are largely incapable of migrating into the lymph node parenchyma.

Since neutrophil recruitment to draining lymph nodes had been reported, following infections with *P. aeruginosa* [21,22] we also investigated popliteal lymph nodes of mice that were s.c. treated with heat-inactivated *P. aeruginosa* for 24 h. Interestingly, numerous endogenous neutrophils were found within the enlarged popliteal lymph node. Applying anti-CD11b and anti-Ly6G mAb immunohistology revealed a massive infiltration of endogenous neutrophils within the medulla as well as the B cell follicle and also partially within the T cell zone (Figure 3C). In this setup, it seems most likely that endogenous neutrophils enter the inflamed lymph nodes from the blood.

### 3.4. Co-Transfer of Neutrophils Together with Dendritic Cells

In earlier studies, we reported that i.l. transferred dendritic cells can enter the lymph node parenchyma by directly migrating through the floor of the subcapsular sinus towards the T cell zone. In contrast, i.l. injected naïve T cells entered the lymph node T cell zone via the medullary region while they failed to directly migrate from the subcapsular sinus into that area. However, once naïve T cells were co-transferred with dendritic cells (DCs) they also could take the direct route [17]. Since i.l. delivered neutrophils are also excluded from entering via the subcapsular sinus floor we next co-injected neutrophils from bone marrow together with in vitro differentiated DCs.

**Figure 4:**
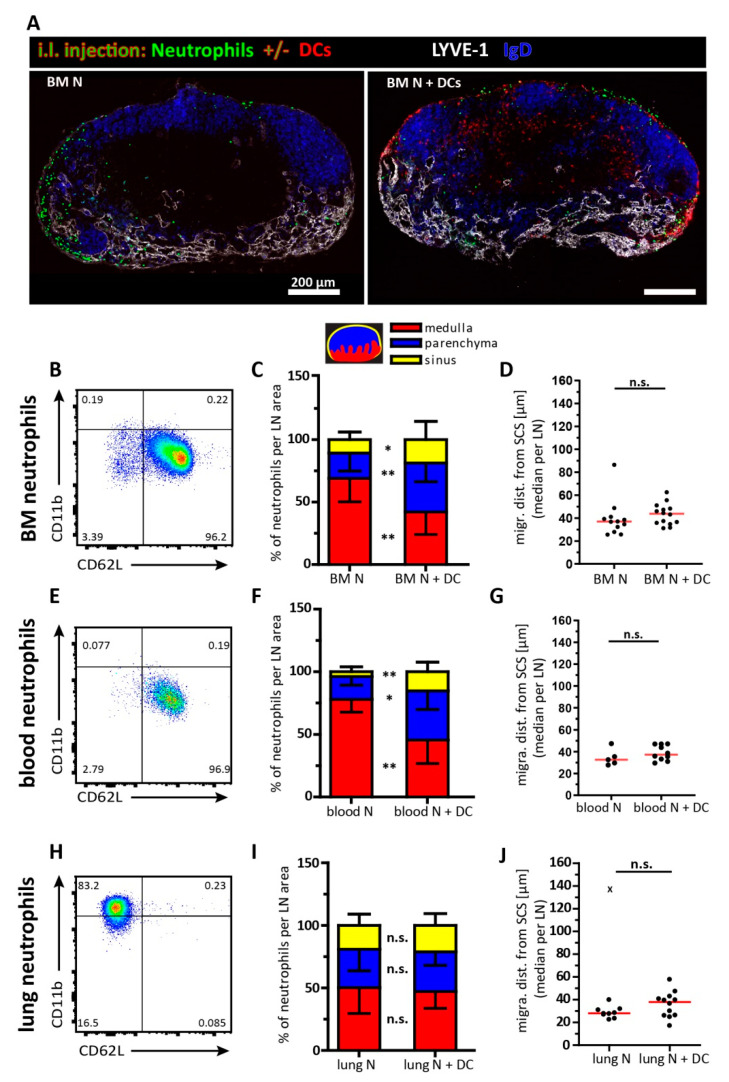
Positioning and migration of bone marrow, blood and lung neutrophils in co-injections with DCs. (**A**) Cryosections of popliteal lymph nodes 4 h after i.l. injection of bone marrow neutrophils (green) alone (left picture) or together with DCs (red; right picture). Lymph node sections were stained with the following antibodies: anti-LYVE-1 (white) and anti-IgD (blue). Scale bar 200 µm. Representative data of 10–12 lymph nodes per group are shown. (**B**) FACS plot showing the activation status of bone marrow neutrophils (CD11b^+^ Ly6G^+^). Representative data of more than 3 independent experiments are shown. (**C**) Distribution of bone marrow neutrophils in lymph node areas: mean and SD of medulla (** *p* = 0.0016), parenchyma (** *p* = 0.0022) and sinus (* *p* = 0.0373). A total of 4 sections per lymph node were analyzed using the Mann–Whitney test. (**D**) Median migration distance of bone marrow neutrophils from the subcapsular sinus per lymph node. Dots, lymph nodes; red line, median; Mann–Whitney test; *p* = 0.1729. Data were pooled from 10–12 different lymph nodes. (**E**) FACS plot displaying the activation status of blood neutrophils (CD11b^+^ Ly6G^+^) derived from an MVA–infected mouse (intranasal infection one day before cell isolation). Representative data of 3 independent experiments are shown. (**F**) Distribution of blood neutrophils in lymph node areas: mean and SD of medulla (** *p* = 0.008), parenchyma (* *p* = 0.0193) and sinus (** *p* = 0.0047). A total of 4 sections per lymph node analyzed. Mann–Whitney test. (**G**) Median migration distance of blood neutrophils from the subcapsular sinus per lymph node. Dots, lymph nodes; red line, median; Mann–Whitney test, *p* = 0.3097; data were pooled from 5–10 different lymph nodes. (**H**) Representative FACS plot with the activation status of lung neutrophils (CD11b^+^ Ly6G^+^) derived from an MVA–infected mouse (intranasal infection one day before cell isolation). Representative data of 3 independent experiments are shown. (**I**) Distribution of lung neutrophils in lymph node areas: mean and SD of medulla (n.s.; *p* = 0.6209), parenchyma (n.s.; *p* = 0.7667) and sinus (n.s.; *p* = 0.4099). A total of 4 sections per lymph node were analyzed using the Mann–Whitney test. (**J**) Median migration distance of lung neutrophils from the subcapsular sinus per lymph node. Dots, lymph nodes; red line, median; Mann–Whitney test, *p* = 0.4483 data were pooled from 10–12 different lymph nodes; x = statistical outlier identified by Grubbs’ test.

Injected alone, bone marrow neutrophils were found in the medullary area and the subcapsular sinus and few were present in the lymph node parenchyma. (Figure 4A, left). Since bone marrow neutrophils did not show an activated phenotype (Figure 4B) this finding confirms the results described above. However, once transferred together with activated DCs, neutrophils primarily positioned at the subcapsular sinus region 4 h after transfer. Several neutrophils are capable to enter the parenchyma by entering through the floor of the sinus (Figure 4A, right). The distribution of neutrophils within the lymph node compartments (medulla, parenchyma and subcapsular sinus) was significantly different upon co-injection with DCs. Here, neutrophils localized more to the subcapsular sinus and the parenchyma compared to the situation without co-injection of DCs (Figure 4C). However, the average migration distances of all neutrophils positioned within the parenchyma were not significantly altered when comparing data from individual lymph nodes (Figure 4D).

Since the migration behavior of neutrophils might also be influenced by their activation and maturation status, we next isolated neutrophils from the blood and lungs of mice that had been intranasally treated with MVA 1 day earlier. As described for the bone marrow-derived neutrophils, neutrophils isolated from blood did also show a resting phenotype (Figure 4E). Likewise, when co-transferred with DCs the distribution of neutrophils throughout the popliteal lymph node was significantly shifted towards the subcapsular sinus and parenchyma (Figure 4F). Although co-injection of DCs allowed neutrophils to enter the lymph node parenchyma more frequently there was no overall increased translocation of these cells towards the deep T cell zone (Figure 4G). In contrast to blood or bone marrow neutrophils, those isolated from MVA-infected lungs showed an activated phenotype reflected by the absence of L-selectin (CD62L) but upregulated levels of CD11b (Figure 4H). Compared to their counterpart from the blood, bone marrow activated neutrophils from the lung were less frequently found in the lymph node parenchyma following their adoptive i.l. transfer. Interestingly, the distribution of neutrophil localization between medulla, parenchyma and sinus did not further change by co-injection of DCs (Figure 4I). Similarly, the average migration distance of neutrophils from the subcapsular sinus towards the lymph node parenchyma was not significantly altered when co-injecting DCs (Figure 4J). Together, these data suggest that the presence of DCs influences the positioning of resting but not of activated neutrophils arriving via afferent lymphatics.

## 4. Discussion

Immune cells gain access to lymph nodes via two different routes: from the blood via specialized high endothelial venules (HEVs) and from peripheral tissues via afferent lymphatics. These two pathways are not equally used by the different immune cell subsets. Recirculating naïve T cells [17] and B cells are known to enter lymph nodes at high frequencies via HEV. In contrast, tissue-resident DCs, once activated migrate towards and subsequently into terminal lymphatic vessels, a process that is facilitated by the expression of the chemokine receptor CCR7 on DCs and its ligands CCL19 and CCL21 by lymphatic endothelial cells [17,23]. Within the terminal lymphatics, DCs migrate towards larger collecting vessels and once those are reached, they passively get transported with the lymph fluid into the subcapsular sinus of the draining lymph node. From there, DCs manage to exit by crawling through preformed pores in the subcapsular sinus floor and move with straight directionality into the T cell zone [17]. Both exit from the subcapsular sinus as well as directional migration again depends on CCR7 and its ligands.

Recent studies suggest that, besides DCs and macrophages, also neutrophils might be able to take up antigen and migrate from the infected tissue site to draining lymph nodes [5,6,12,21,24,25]. However, the frequency of this process, the route by which neutrophils entered these lymph nodes and their localization within lymph nodes remain largely elusive. Therefore, the present study aimed to investigate the positioning of neutrophils in lymph nodes upon homing via lymphatic vessels. We used i.l. transfer of cells, a method developed earlier by our group, in combination with lymph node imaging to address some of these points. The results of this study revealed that within non-inflamed lymph nodes i.l. transferred neutrophils localized primarily to the medulla or the subcapsular sinus. Even following lymph node inflammation, neutrophils stayed primarily in the subcapsular sinus or within the medulla and only rarely entered the lymph node parenchyma. Interestingly, resting neutrophils entered to some degree the interfollicular area via the subcapsular sinus floor upon co-injection with DCs while in vivo-activated neutrophils were also capable to enter to some degree the interfollicular area even in the absence of DCs.

Neutrophils are known to circulate in the blood [26] and to migrate into the tissue upon infection or inflammation. Based on the experimental setup applied in this study, adoptively transferred neutrophils were passively transported with the lymph flow into the subcapsular sinus of the draining lymph node. Some of the transferred cells stayed for several hours at this location, while others were found at later time points within the medulla. This behavior was similar to the situation observed before for naïve T cells within the first two hours of i.l. delivery. However, in contrast to the situation for naïve T cells that were able to translocate from the medullary region into the T cell zone during the following 2 h [17] neutrophils failed to translocate into the deep T cell zone. Our earlier finding that i.l. transferred naïve, CCR7-deficient T cells were also excluded from the T cell zone suggested that expression of CCR7 is indispensable for this process. We therefore checked whether any of the neutrophil populations used in this study—resting or activated—express CCR7. However, as reported by others [13], we failed to find any evidence that neutrophils express this lymph node homing chemokine receptor (data not shown). Furthermore, the absence of inflammatory stimuli and thus lack of inflammatory chemokines in non-inflamed lymph nodes could also contribute to impaired neutrophil migration into the lymph node parenchyma.

It was shown by others that Phorbol-12-myristate-13-acetate (PMA) serves as a potent chemical activator of neutrophils [27]. We were able to confirm the potential of this agent to strongly stimulate neutrophils (data not shown). Neutrophil activation was also described upon recognition of various pathogenic particles or pathogen-derived molecules via pattern recognition receptors [2]. Results obtained in the present work confirmed that all of the tested pathogens have the potential to activate neutrophils. Activated neutrophils are known to express high levels of CD11b [26] which was also confirmed in the present study. Interestingly, Hampton et al. showed a CD11b-dependent migration of neutrophils via lymphatic vessels to lymph nodes [6]. However, the distribution of neutrophils within these lymph nodes had not been investigated in that study. Results of the present study showed that in vitro-activated CD11b^high^ neutrophils migrated occasionally into the lymph node parenchyma. Nevertheless, the majority of these highly-activated i.l. transferred neutrophils were retained in the subcapsular sinus. Since PMA is known as a potent activator of granule release [27], it seems possible that neutrophils attached to each other due to their strong activation and granule release. Therefore, strong in vitro-activation, which was revealed in the present study, might have contributed to rapid neutrophil accumulation within the subcapsular sinus. This accumulation of neutrophils or formation of cell aggregates might prevent further migration and thus neutrophils might get stuck within the subcapsular sinus for prolonged periods of time. The present study also showed that the localization within the lymph node compartments is similar for resting and activated neutrophils. As mentioned above, the lack of neutrophil chemo-attractants in non-inflamed lymph nodes might be a factor that prevents neutrophil translocation to the deep T cell zone. This model is in line with the idea that pre-activated cells, such as some of the neutrophils used in the present study, still require chemo-attractant signals provided by the environment in order to directional migrate within tissues.

Massive neutrophil lymph node infiltration upon s.c. infection with *P. aeruginosa* was recently reported by others [21,22]. Kastenmüller et al. showed that endogenous neutrophils localized within the subcapsular sinus, the medulla as well as interfollicular areas. Results of the present study confirmed that presumably blood-derived neutrophils infiltrated the draining lymph node after the s.c. application of heat-inactivated *P. aeruginosa*. Here, blood-derived neutrophils migrated into the lymph node parenchyma of *P. aeruginosa*-activated lymph nodes, whereas intra-lymphatically transferred neutrophils failed in doing so. The reasons for these differences are currently not clear. Endogenous neutrophils are released from the bone marrow to the blood to migrate into infected tissue and some of them might reach the draining lymph node by reverse migration. In contrast, i.l. transferred neutrophils were isolated from the bone marrow and subsequently injected into the lymph vessel of the draining lymph node. Therefore, it seems likely that bone marrow neutrophils were not primed or “mature” enough to migrate into the lymph node parenchyma, as endogenous neutrophils could do after being activated at the site of infection. Another possibility could be that activating signals mediated from the lymphatic endothelial cells were not potent enough for i.l. delivered neutrophils to migrate into the lymph node parenchyma. Alternatively, the s.c. injection of heat-inactivated bacteria might lead to the induction of chemotactic molecules on HEV or other capillary vessels within the lymph node that allows broad access of neutrophils from the blood into the lymph node parenchyma without any further translocation.

Surprisingly, this study also revealed that i.l. neutrophils that were isolated from inflamed lungs are capable to enter interfollicular areas of lymph nodes. The presence of DCs neither affected their distribution within the lymph node nor facilitated their translocation deeper into the parenchyma. In contrast to bone marrow and blood neutrophils, the lung neutrophils used in the present study were highly activated, reflected by high levels of CD11b and the absence of CD62L expression. In particular, increased expression of the adhesion molecule CD11b and other factors present in activated but not in resting neutrophils such as proteases might facilitate their egress from the subcapsular sinus floor.

Data from the present study revealed a change in the localization of resting bone marrow and blood neutrophils upon co-transfer with DCs. Neutrophils co-injected with DCs positioned in the subcapsular sinus, and subsequently migrated to some extent into the lymph node parenchyma, whereas neutrophils injected alone were less proficient in exiting from the subcapsular sinus. This observation suggests that the presence of co-migrating DCs affects the behavior of bone marrow and blood neutrophils. We reported earlier that migrating DCs might induce changes in the subcapsular sinus floor that allowed direct homing of naïve T cells which usually entered the lymph node parenchyma in a retrograde manner via the medullary sinuses [17]. This model would also help to explain the finding of the present study that co-delivered DCs supported neutrophils to enter the lymph node parenchyma via such potential changes. More recently we reported preformed pores within the subcapsular sinus floor [28]. Such preformed pores could have been potentially expanded by co-migrating DCs, and therefore might also allow neutrophils to enter the lymph node parenchyma directly from the subcapsular sinus. Another explanation for neutrophil entry could be that co-injected DCs block the subcapsular sinus lumen and therefore trapped smaller cells, such as neutrophils in this area. This might give neutrophils more time to find potential entry pores in the subcapsular sinus floor to subsequently enter the lymph node parenchyma.

In the present study, we addressed the migration and positioning of isolated neutrophils in draining lymph nodes following their i.l. delivery. Although this process has an artificial component since it circumvents the need for neutrophils to enter from tissue via lymphatic capillaries into lymphatic vessels, it offers the opportunity to manipulate and dissect several aspects of neutrophil homing to lymph nodes and subsequent positioning. This approach revealed that lymph-derived neutrophils overall locate primarily to the subcapsular and medullary sinus system while they rarely entered the deep T cell zone. The absence of mouse neutrophils from this lymph node area goes along with the lack of detectable expression of surface CCR7 on neutrophils in the present study. However, intracellular stores of CCR7 in mouse neutrophils were described by others [11] while experiments from the present study indicate that those, if present, did not functionally translocate to the cell surface in order to allow neutrophil entry into the T cell zone. The absence of neutrophils from the T cell zone goes along with the inability of murine neutrophils to activate T cells. The observations made in the mouse model are however different from the situation in humans. In the latter species, a subset of neutrophils expresses MHC-II and co-stimulatory molecules as well as the T zone homing chemokine receptor CCR7 [29]. These molecules get in particular upregulated after exposure to antigen-immune globulin complexes and allow activation of CD4^+^ T cells [30]. In the human lymph node, these cells locate to the interfollicular area, a place known for rapid T cell activation [30]. Together these data indicate that lymph node neutrophils serve a different function in different species.

In conclusion, the present study did not aim to address whether and under which conditions neutrophils are able to migrate from inflamed tissue via afferent lymphatics to draining lymph nodes. Instead, we used i.l. cell delivery to study whether the activation status of the neutrophils or that of the draining lymph node affects the positioning of the adoptively transferred cells. Although we observed variations to some degree most of the transferred cells were either found in the subcapsular sinus or in the medulla irrespective of their activation and only very few cells were found in the T or B cell areas. However, inflammatory stimuli led to substantial recruitment of neutrophils from blood into the lymph node parenchyma but the role of lymph-derived neutrophils in lymph node physiology further remains elusive.

## Data Availability

Data will be made available on reasonable request.

## Supplementary Materials

The following are available online at https://www.mdpi.com/article/10.3390/cells10061486/s1, Video S1: Neurophil motility and migration behavior in healthy tissue; Video S2: Neutrophil motility and migration behavior shortly after induction of a sterile injury.

## Abbreviations

CCR7	CC chemokine receptor 7
CCL19	CC-chemokine ligand 19
CCL21	CC-chemokine ligand 21
CD11b	Integrin α-M
CXCR4	CXC-Motive-Chemokinereceptor 4
CD62L	L-selectin
DCs	dendritic cells
HEVs	high endothelial venules
LTB4	Leukotriene B4
LYVE1	Lymphatic Vessel Endothelial Hyaluronan Receptor 1
PRRs	pattern recognition receptors
PMA	Phorbol-12-myristate-13-acetate
TLR4	Toll-like receptor 4

## References

[1] Mayadas T.N., Cullere X., Lowell C.A. (2014). The Multifaceted Functions of Neutrophils. Annu. Rev. Pathol. Mech. Dis..

[2] De Oliveira S., Rosowski E., Huttenlocher A. (2016). Neutrophil migration in infection and wound repair: Going forward in reverse. Nat. Rev. Immunol..

[3] Nauseef W.M., Borregaard N. (2014). Neutrophils at work. Nat. Immunol..

[4] Girbl T., Lenn T., Perez L., Rolas L., Barkaway A., Thiriot A., del Fresno C., Lynam E., Hub E., Thelen M. (2018). Distinct Compartmentalization of the Chemokines CXCL1 and CXCL2 and the Atypical Receptor ACKR1 Determine Discrete Stages of Neutrophil Diapedesis. Immunity.

[5] Abadie V., Badell E., Douillard P., Ensergueix D., Leenen P.J.M., Tanguy M., Fiette L., Saeland S., Gicquel B., Winter N. (2005). Neutrophils rapidly migrate via lymphatics after Mycobacterium bovis BCG intradermal vaccination and shuttle live bacilli to the draining lymph nodes. Blood.

[6] Hampton H.R., Bailey J., Tomura M., Brink R., Chtanova T. (2015). Microbe-dependent lymphatic migration of neutrophils modulates lymphocyte proliferation in lymph nodes. Nat. Commun..

[7] Rigby D.A., Ferguson D.J.P., Johnson L.A., Jackson D.G. (2015). Neutrophils rapidly transit inflamed lymphatic vessel endothelium via integrin-dependent proteolysis and lipoxin-induced junctional retraction. J. Leukoc. Biol..

[8] Teijeira A., Halin C. (2015). Editorial: Breaching their way through: Neutrophils destroy intercellular junctions to transmigrate rapidly across lymphatic endothelium. J. Leukoc. Biol..

[9] Maletto B.A., Ropolo A.S., Alignani D.O., Liscovsky M.V., Ranocchia R.P., Moron V.G., Pistoresi-Palencia M.C. (2006). Presence of neutrophil-bearing antigen in lymphoid organs of immune mice. Blood.

[10] Bogoslowski A., Butcher E.C., Kubes P. (2018). Neutrophils recruited through high endothelial venules of the lymph nodes via PNAd intercept disseminating Staphylococcus aureus. Proc. Natl. Acad. Sci. USA.

[11] Beauvillain C., Cunin P., Doni A., Scotet M., Jaillon S., Loiry M.-L., Magistrelli G., Masternak K., Chevailler A., Delneste Y. (2011). CCR7 is involved in the migration of neutrophils to lymph nodes. Blood.

[12] Hampton H.R., Chtanova T. (2016). The lymph node neutrophil. Semin. Immunol..

[13] Gorlino C.V., Ranocchia R.P., Harman M.F., García I.A., Crespo M.I., Morón G., Maletto B.A., Pistoresi-Palencia M.C. (2014). Neutrophils Exhibit Differential Requirements for Homing Molecules in Their Lymphatic and Blood Trafficking into Draining Lymph Nodes. J. Immunol..

[14] Yung L.-Y.L., Lim F., Khan M.M., Kunapuli S.P., Rick L., Colman R.W., Cooper S.L. (2000). High-molecular-weight kininogen preadsorbed to glass surface markedly reduces neutrophil adhesion. Biomaterials.

[15] Halle S., Dujardin H.C., Bakocevic N., Fleige H., Danzer H., Willenzon S., Suezer Y., Hämmerling G., Garbi N., Sutter G. (2009). Induced bronchus-associated lymphoid tissue serves as a general priming site for T cells and is maintained by dendritic cells. J. Exp. Med..

[16] Bakočević N., Worbs T., Davalos-Misslitz A., Förster R. (2009). T Cell–Dendritic Cell Interaction Dynamics during the Induction of Respiratory Tolerance and Immunity. J. Immunol..

[17] Braun A., Worbs T., Moschovakis G.L., Halle S., Hoffmann K., Bölter J., Münk A., Förster R. (2011). Afferent lymph–derived T cells and DCs use different chemokine receptor CCR7–dependent routes for entry into the lymph node and intranodal migration. Nat. Immunol..

[18] Mantovani A., Cassatella M.A., Costantini C., Jaillon S. (2011). Neutrophils in the activation and regulation of innate and adaptive immunity. Nat. Rev. Immunol..

[19] Sagoo P., Garcia Z., Breart B., Lemaitre F., Michonneau D., Albert M.L., Lévy Y., Bousso P. (2015). In vivo imaging of inflammasome activation reveals a subcapsular macrophage burst response that mobilizes innate and adaptive immunity. Nat. Med..

[20] Abadie V., Bonduelle O., Duffy D., Parizot C., Verrier B., Combadière B. (2009). Original Encounter with Antigen Determines Antigen-Presenting Cell Imprinting of the Quality of the Immune Response in Mice. PLoS ONE.

[21] Lämmermann T., Afonso P.V., Angermann B.R., Wang J.M., Kastenmüller W., Parent C.A., Germain R.N. (2013). Neutrophil swarms require LTB4 and integrins at sites of cell death in vivo. Nat. Cell Biol..

[22] Kastenmüller W., Torabi-Parizi P., Subramanian N., Lämmermann T., Germain R.N. (2012). A Spatially-Organized Multicellular Innate Immune Response in Lymph Nodes Limits Systemic Pathogen Spread. Cell.

[23] Eckert N., Permanyer M., Yu K., Werth K., Förster R. (2019). Chemokines and other mediators in the development and functional organization of lymph nodes. Immunol. Rev..

[24] Kamenyeva O., Boularan C., Kabat J., Cheung G.Y.C., Cicala C., Yeh A.J., Chan J.L., Periasamy S., Otto M., Kehrl J.H. (2015). Neutrophil Recruitment to Lymph Nodes Limits Local Humoral Response to Staphylococcus aureus. PLoS Pathog..

[25] Chtanova T., Schaeffer M., Han S.-J., van Dooren G.G., Nollmann M., Herzmark P., Chan S.W., Satija H., Camfield K., Aaron H. (2008). Dynamics of Neutrophil Migration in Lymph Nodes during Infection. Immunity.

[26] Kolaczkowska E., Kubes P. (2013). Neutrophil recruitment and function in health and inflammation. Nat. Rev. Immunol..

[27] Boxio R., Bossenmeyer-Pourié C., Steinckwich N., Dournon C., Nüße O. (2003). Mouse bone marrow contains large numbers of functionally competent neutrophils. J. Leukoc. Biol..

[28] Martens R., Permanyer M., Werth K., Yu K., Braun A., Halle O., Halle S., Patzer G.E., Bošnjak B., Kiefer F. (2020). Efficient homing of T cells via afferent lymphatics requires mechanical arrest and integrin-supported chemokine guidance. Nat. Commun..

[29] Vono M., Lin A., Norrby-Teglund A., Koup R.A., Liang F., Loré K. (2017). Neutrophils acquire the capacity for antigen presentation to memory CD4+ T cells in vitro and ex vivo. Blood.

[30] Lok L.S.C., Dennison T.W., Mahbubani K.M., Saeb-Parsy K., Chilvers E.R., Clatworthy M.R. (2019). Phenotypically distinct neutrophils patrol uninfected human and mouse lymph nodes. Proc. Natl. Acad. Sci. USA.

